# Host Defence Peptides: A Potent Alternative to Combat Antimicrobial Resistance in the Era of the COVID-19 Pandemic

**DOI:** 10.3390/antibiotics11040475

**Published:** 2022-04-01

**Authors:** Waqas Ali, Ahmad Elsahn, Darren S. J. Ting, Harminder S. Dua, Imran Mohammed

**Affiliations:** Section of Ophthalmology, Larry A. Donoso Laboratory for Eye Research, Academic Unit of Mental Health and Clinical Neuroscience, School of Medicine, University of Nottingham, Queens Medical Centre, Eye and ENT Building, Nottingham NG7 2UH, UK; mzywa1@nottingham.ac.uk (W.A.); elsahn@me.com (A.E.); ting.darren@gmail.com (D.S.J.T.); harminder.dua@nottingham.ac.uk (H.S.D.)

**Keywords:** host defence peptides, antimicrobial resistance, COVID-19, antimicrobial peptides, immunomodulatory peptides

## Abstract

One of the greatest challenges facing the medical community today is the ever-increasing trajectory of antimicrobial resistance (AMR), which is being compounded by the decrease in our antimicrobial armamentarium. From their initial discovery to the current day, antibiotics have seen an exponential increase in their usage, from medical to agricultural use. Benefits aside, this has led to an exponential increase in AMR, with the fear that over 10 million lives are predicted to be lost by 2050, according to the World Health Organisation (WHO). As such, medical researchers are turning their focus to discovering novel alternatives to antimicrobials, one being Host Defence Peptides (HDPs). These small cationic peptides have shown great efficacy in being used as an antimicrobial therapy for currently resistant microbial variants. With the sudden emergence of the SARS-CoV-2 variant and the subsequent global pandemic, the great versatility and potential use of HDPs as an alternative to conventional antibiotics in treating as well as preventing the spread of COVID-19 has been reviewed. Thus, to allow the reader to have a full understanding of the multifaceted therapeutic use of HDPs, this literature review shall cover the association between COVID-19 and AMR whilst discussing and evaluating the use of HDPs as an answer to antimicrobial resistance (AMR).

## 1. Introduction

In December 2019, the World Health Organisation (WHO) was made aware of a cluster of cases of pneumonia in Wuhan, the People’s Republic of China (PRC), caused by a novel coronavirus (nCov) [1]. This coronavirus was distinct and different yet structurally similar to viruses that were responsible for previous pandemics such as severe acute respiratory syndrome (SARS-CoV) and Middle East respiratory syndrome (MERS-CoV) [2]. Zhu and co-workers isolated and characterised this virus as 2019-nCoV, which later was renamed SARS-CoV-2 or COVID-19 [2,3]. This virus rapidly spread from its reported origin in Wuhan to other parts of the world, leading to the WHO declaring a pandemic in March 2020. The COVID-19 pandemic has, to date, transformed the world we live in by affecting all aspects of human life, from psychosocial to financial aspects and beyond, leaving fears that the full extent of repercussions is to be felt in the near and distant future [4].

### 1.1. Virus Classification and Structure

The SARS-CoV-2 virus belongs to the family of *coronaviridae* and so takes its place next to other coronaviruses such as MERS-CoV and SARS-CoV due to its close taxonomy as well as similarities in morphology and chemical structure. All coronaviruses are single-stranded RNA viruses sized 80–220 nm in diameter and have a thick envelope [5]. From the surface of the envelope, glycoproteins that resemble club-shape spikes protrude and under electron microscopy resemble the corona of the sun and are therefore given the name coronavirus [5,6]. It is these glycoproteins which are responsible for the virus attaching to the host cell as well as carrying the main antigenic epitopes which are a target site for neutralising antibodies that recognise that site [7]. The single strand of RNA is found within a capsid and is associated with a nucleoprotein. What makes the SARS-CoV-2 virus distinct from other coronaviruses is the nucleotide sequence which, after whole-genome sequencing, showed 79.0% and 51.8% similarity to SARS-CoV and MERS-CoV, respectively [8,9].

### 1.2. Virus Transmission and Disease Pathogenesis

The SARS-CoV-2 virus has several different transmission routes from person to person such as respiratory droplets and contact transmission, with the former being considered the main transmission route [10]. Large respiratory droplets formed via the host breathing, sneezing and coughing may contain the virion which, when coming into contact, could contaminate inanimate surfaces (fomites). These fomites play a role in transmission via person-to-person contact as the virion can remain on surfaces for as long as 72 h [11].

**Figure 1:**
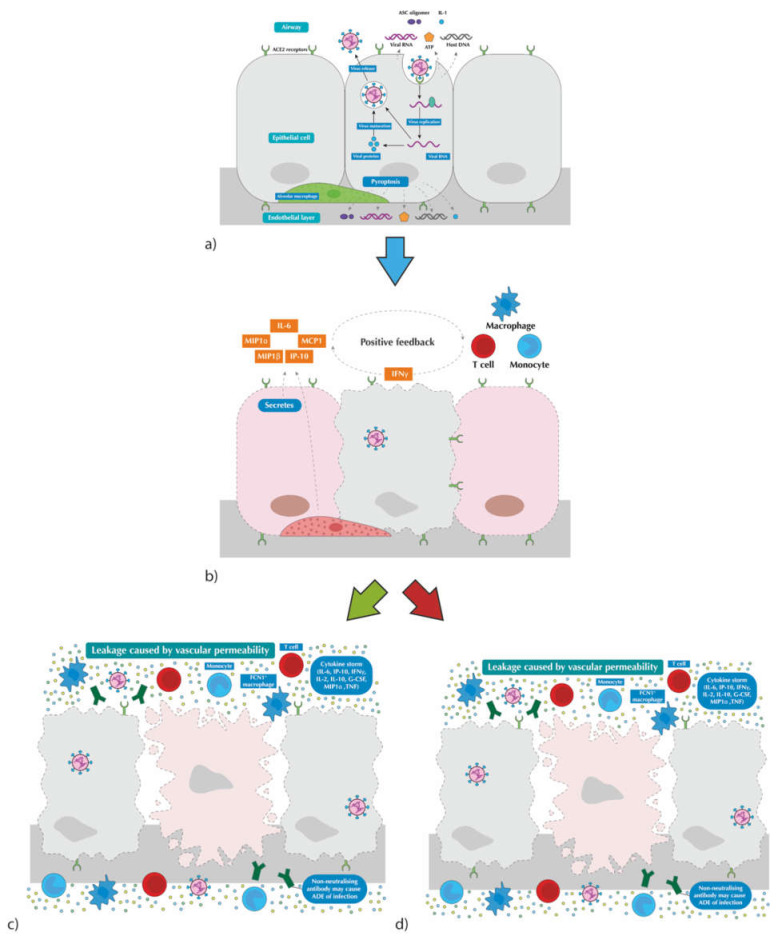
Pathogenesis of COVID-19. (**a**) Following the infiltration of host cell, SARS-CoV-2 undergoes replication and maturation leading to pyroptosis and release of damage-causing molecules; (**b**) secretion of pro-inflammatory cytokines and chemokines recruit immune cells, eventually setting up a positive feedback loop; (**c**) activated T-cells eradicates infected cells; (**d**) dysfunctional immune responses cause an overproduction of cytokines resulting in a damaging ‘cytokine storm’ leading to widespread inflammation and bystander tissue damage. (edited) [13].

Extensive research has been carried out showing that SARS-CoV-2 virus principally targets cells via binding to angiotensin-converting enzyme 2 (ACE2) receptor within the respiratory epithelium, alveolar epithelium and vascular endothelial in addition to macrophages in the lung [12]. Once it is internalised, the virion undergoes active replication and virus maturation, which eventually damages the cells to cause cell pyroptosis, leading to the release of damage-causing molecules such as ATP and inflammasome oligomers. [13]. These molecules are recognised by adjacent epithelial cells and macrophages, causing an inflammatory cascade, with the secretion of pro-inflammatory cytokines and chemokines (IL-6, IP-10, MIP etc.) allowing for recruitment of inflammatory cells, namely T-Cells and monocytes [14]. Once the inflammatory cells are at the site of infection, further secretion of inflammatory proteins can lead to a pro-inflammatory feedback loop. A healthy immune response will attract virus-specific T-cells that will eliminate the viral cells and mediate an efficient immune response, namely the secretion of virus-neutralising antibodies. Alongside this, macrophages will recognise the neutralised virus cells and apoptotic cells and clear them by phagocytosis, leading to a quick response with minimal lung infrastructural damage [13]. The greatest concern is the dysfunctional immune response within patients who have SARS-CoV-2 and underlying risk factors causing an impaired immune system or even immunodeficiency. In this scenario, the recruitment of immune cells can cause an over-production of pro-inflammatory cytokines, resulting in a cytokine storm that leads to widespread inflammation and multi-organ damage, which in extreme cases leads to death (see Figure 1) [14,15].

Following the initial infiltration of SARS-CoV-2 it can take up to 0 to 14 days for the virus to intubate and the body to show symptoms [16]. Due to the multi-facets of the body’s immune response, SARS-CoV-2 shows a varied degree of severity in clinical manifestation. Research on previous cases of SARS-CoV-2 have found that severity of the disease can be divided into four categories: mild, moderate, severe and critical, with each category showing distinct symptoms (Table 1) [17].

### 1.3. Management of COVID-19

As with all novel diseases, initial treatment is mainly based on the clinician’s experience in dealing with similar diseases previously encountered and in this instance the MERS-CoV and SARS-CoV paved the way. COVID-19, however, posed a problem as there was no specific treatment for the previous pandemics. The treatment aims were to relieve symptoms and thus far there is no empirical treatment for SARS-CoV-2 available. Initially, a patient who has contracted SARS-CoV-2 is isolated to prevent the spread to others. Whilst treatment for the mild symptoms is not needed, patients presenting with breathing difficulties are placed on supplemental oxygen and may be started on an oral regimen of corticosteroids such as dexamethasone to alleviate the inflammatory response [18].

Currently, the National Institute for Health and Care Excellence (NICE), United Kingdom has investigated antibiotic use for patients with pneumonia in hospitals and produced guidance for medical practitioners [19]. Emphasis has particularly been given to the clinical judgement of medical practitioners when prescribing antibiotics to patients, only advising antibiotic use when there is evidence of bacterial infection [19]. In choosing the most suitable antibiotics to use, the medical practitioner must take into account the type of pneumonia, whether it is community-acquired or hospital-acquired (Table 2 and Table 3) [20].

Extensive research has shown that, as with many pulmonary diseases of viral origin, there is a high risk of manifestation of secondary bacterial infections which can lead to serious complications and even death [21]. By looking at previous trends in pandemics such as SARS-CoV, over 30% of patients were diagnosed with secondary bacterial infections and this was highly correlated to the disease severity [21]. Extensive research has been carried out to measure the prevalence and severity of secondary bacterial infections of SARS-CoV-2 patients, with studies ranging from low prevalence to other studies citing high prevalence and whether empiric use of antibiotics is beneficial. Yet with all these studies, the question remains on the use of antibiotics for SARS-CoV-2 and associated symptoms [14,22,23,24]. Clinicians must therefore weigh up the benefit of antibiotic use without adding to the emerging pandemic of antimicrobial resistance.

## 2. Antimicrobial Agents

Antimicrobial agents such as antibiotics are widely used for the management of bacterial infections. Antibiotics either prevent the growth of bacteria (bacteriostatic) allowing for the body’s immune system to produce an effective response to target the infection, or have mechanisms which kill the bacterial cell (bactericidal) [25]. The mechanisms of action of these two classes of antibiotics are not exclusive as a bactericidal antibiotic may have bacteriostatic activity at low concentrations [25].

From the first antibiotic discovery, notably penicillin, antibiotics have been hailed as a wonder drug, allowing clinicians to successfully carry out procedures which would otherwise have been fatal due to bacterial infections [26]. As such, the success of antibiotics is truly remarkable; however, this may be overshadowed by their side effects, namely toxicity and increase in secondary infections due to disturbance of normal flora.

### 2.1. Toxicity

Toxicity was previously thought to be a specific biomedical property of a drug; however, with the progression in the field of toxicology the definition is now more of a descriptor of the adverse effects of the drug during drug–host interaction [27].

Studying the relationships between different drugs and how they interact with the body can give crucial information on how drugs can be used to their full potential and how toxicity can be limited. Stereoisomerism of the compound has been shown to affect the pharmacodynamics, pharmacokinetics and toxicity of the drug [28]. One example of an antibiotic having stereoisomerism is levofloxacin, which is a L-enantiomer of the racemic ofloxacin [29]. Studies have shown that D-ofloxacin has significantly lower antibacterial activity compared to levofloxacin yet is more toxic, resulting in an increased risk of seizures and phototoxicity [30]. Other unforeseeable idiosyncratic reactions may be due to the synthesis of chemically reactive metabolites [31]. These reactive metabolites can mediate adverse reactions and/or toxicity. For example, quinoneimine, the unstable metabolite of an antimalarial agent called amodiaquine, was shown to be responsible for the development of life-threatening neutropenia and hepatotoxicity [30,32].

With the reduction of our antibiotic armamentarium, researchers have looked into approaches such as increasing antibiotic drug dose to achieve a better clinical response [33]. Over-dosages are not regular clinical practice; however, there are some notable examples such as the treatment of infective endocarditis and penicillin-resistant pneumococci, whereby penicillin is administered above the recommended dosage [33]. Nevertheless, extensive research has found that most antibiotic-related toxicity is caused by administration of high dose and long-term administration of antibiotics (see Table 4) [34].

### 2.2. Antimicrobial Resistance

Indiscriminate and inappropriate use of antimicrobials such as antibiotics is exacerbating the already volatile situation of global antimicrobial resistance (AMR). According to the WHO, by 2050, AMR is predicted to claim over 10 million lives and reduce the global GDP by 2 to 3.5% with a loss of 100 trillion USD [35].

Healthcare systems across the world are reliant on the use of antimicrobials from prophylactic use in surgical interventions to treatment of local and systemic pathogenic infections. Effective treatment of conditions such as organ transplantation, intensive care for pre-term new-borns and chemotherapy for cancer patients cannot be carried out without effective antimicrobials [36]. Research carried out highlights the necessity of using antimicrobials to save lives and the lack of antibiotics result in more deaths than antibiotic resistance [37]. Therefore, healthcare systems must use antimicrobials with caution and sparingly, using them only in the presence of appropriate indications. Another factor is the use of antimicrobials in agriculture and cultivation. Mass use of antimicrobials in food production and mass prophylaxis in animals to prevent infections and aid in growth leads to contamination and the increased frequency of antimicrobial-resistant genes in the local microorganism population [36]. What weaves all the above factors that play a part in AMR is the misuse of antimicrobials, which inevitably leads to resistant microorganisms.

What compounds the effect of AMR is the lack of development of novel antimicrobials. Since the golden age of antibiotic discoveries of the 1970s to 1980s, no new classes of antibiotics have been discovered [38]. The challenge of discovering and developing novel antibiotics is time consuming and can take over 15 years from discovering the active substance in the antibiotics to trailing and testing on a variety of media and hosts [39]. Once tested, the drug must be given regulatory approval by government departments, adding to the time and costs incurred [39]. As a result, pharmaceutical companies are less likely to invest their efforts into such projects, deepening the stagnation of development of novel antimicrobials. According to the WHO, there are currently 41 new antibiotics in different stages of development; however, only a handful of these new antibiotics are suitable for the most common bacterial infections in the community, namely those caused by “ESKAPE” pathogens *(Enterococcus faecium*, *Staphylococcus aureus*, *Klebsiella pneumoniae*, *Acinetobacter baumanii*, *Pseudomonas aeruginosa* and *Enterobacter* species) [40,41].

## 3. Host defence Peptides: A Potential Solution for the Antimicrobial Resistance

Host defence peptides (HDPs), also commonly known as antimicrobial peptides (AMPs), are naturally occurring molecules that show broad-spectrum activities including immunomodulatory, wound-healing, antimicrobial and anticancer [42] properties. Most HDPs are cationic and amphipathic with a size of up to 50 amino acids in length [43]. Due to their cationic (positively charged) nature, they directly bind to the anionic (negatively charged) surface of the bacterial membrane causing lytic cell death [43]. The selectivity of HDPs can be rationally optimised using the structure–activity relationship (SAR) approach. With all this considered, HDPs are increasingly being used as analogues of antibiotics and a suitable alternative to antimicrobials.

### 3.1. History of HDPs

With the discovery and isolation of lysozyme by Alexander Fleming in 1922, the attention of clinicians was drawn to molecules within the body as the first line of defence against pathogenic microorganism, more specifically molecules that played a role in the body’s innate immune system [44]. This innate system, comprised of a barrier surface (i.e., skin and mucosal surfaces), reduced the pH of the stomach and the sweeping motion of the cilia in the airways, including the synergistic effect of chemical molecules such as complement proteins and HDPs, providing the body with a non-specific but rapid response to invading pathogens. In 1939, René Dubos isolated the antimicrobial substance from the soil bacteria, *Bacillus brevis*, which was later called gramicidin. Further investigations showed that gramicidin is a mixture of six HDPs called N-formylated polypeptides [45,46]. Within the animal kingdom, it was discovered that wax moth larvae produced soluble HDPs in response to infection caused by *Pseudomonas aeruginosa* [47], whereas in plants, crystalline protein isolated from wheat flour exhibited antimicrobial properties, which with progressive research were later renamed plant defensins due to their role in enhancing plant response against phytopathogen infections [48,49]. Despite the discoveries of these naturally occurring HDPs, the focus of therapeutics turned towards the new and upcoming antimicrobials, heralding an era of over-reliance on antimicrobials and consequently fuelling the pandemic of AMR. With the focus primarily on the success of antibiotics, HDPs were almost lost in time and only when the dangers of AMR were to be realised and the discovery of mammalian HDPs (e.g., defensins and cathelicidins) in the 1980s, did focus slowly shift back to the use of HDPs [50].

Initially, the method of discovering, isolating, and characterising new HDPs was by chromatographic approaches; however, due to the recognition of peptide-sequence motifs, a more genomic and bioinformatic approach was used [51]. This approach required a central database in which the necessary bio-information could be located, thus kickstarting the creation of multiple data repositories and databases specifically holding information about HDPs such as the antimicrobial peptide database (APD3) and the Data repository of antimicrobial peptides (DRAMP). According to APD3 there are currently 2619 HDPs and according to DRAMP only 76 HDPs are in drug-development phases [52,53].

### 3.2. Structure of HDPs

HDPs are oligopeptides that are biologically produced by many different sources and are distributed throughout the human body [54]. Due to this wide distribution, the structure of HDPs also differs, which led to the antimicrobial peptide databases proposing a classification of HDPs based on their three-dimensional structure [55]. This proposed approach classified natural HDPs into four families: α, β, αβ and non-αβ. HDPs belonging to the α family consisted of a α-helical structure and the β family consisted of at least two β-strands [55]. The HDPs classified in the αβ family had both α-helical structures and β-strands, whereas the non-αβ had neither [55]. HDPs can also be synthesised with flexible loops or extensions [56]. Other classification approaches have been proposed for the ease of identification such as classification based on sources, e.g., mammalian-derived, amphibian derived etc, or classification based on activity, e.g., antibacterial, antiviral, antifungal, etc., and classification based on amino-acid residues, e.g., proline-rich, tryptophan-rich, etc. [43].

Most HDPs are small molecules which usually have fewer than 50 amino acids (see Table 5). These molecules are mainly amphipathic, containing both hydrophobic and hydrophilic parts, and cationic with a net charge between +2 to +9 at physiological pH. The latter characteristics are due to the abundance of Arginine and Lysine residues [57].

In this review, we shall only mention a few commonly studied HDPs that are found within humans.

### 3.3. HDPs Found in Humans

#### 3.3.1. Defensins

Human defensins are highly cationic with approximately 30 amino acids of length. They are broadly classified into two sub-families in humans based on their structure: α- and β-defensins [58,59]. Three α-defensins, named human neutrophil peptides (HNPs), were first isolated in 1985 by the Lehrer group from human neutrophils [60]. To date, the family of human α-defensins comprised of six HDPs: HNP-1 to -4 and human defensin (HD)-5 and -6 [61,62,63]. HNP-1 to -4 are abundantly found in human bone marrow, peripheral blood leukocytes, the spleen and the thymus, whereas HD-5 and HD-6 are tissue specific and can only be found in the Paneth cells of the intestinal epithelium [64]. HNP-1 to -3 were found to be effective at killing *Staphylococcus aureus*, *Pseudomonas aeruginosa* and *Escherichia coli*, whereas HNP-4 was effective at killing *Escherichia coli*, *Streptococcus faecalis* and *Candida albicans* [60,63].

Human β-defensin (hBD)-1 was first isolated from human plasma and the kidney. hBD-1 to -3 have been extensively studied due to their multi-functional properties including being antimicrobial, immunomodulatory, chemotactic and antitumorigenic [65]. HBD-1 to -3 displays potent antimicrobial activity against *Escherichia coli*, *Pseudomonas aeruginosa* and *Candida albicans* [66,67]. HBD-9, also known as DEFB109, was first identified as a pseudogene. We and others have later confirmed that HBD-9 is an active gene and down-regulates during infection caused by bacterial, viral and acanthamoeba keratitis [68,69,70,71,72,73]. Our recent study demonstrated that hBD-9 levels are elevated in samples of patients with fungal keratitis [71].

#### 3.3.2. Histatins

In 1988, histatin 1, 3 and 5 isolated from human saliva showed potent ability to kill *Candida albicans* [74]. These histatins are exclusively expressed in human salivary secretions [75].

#### 3.3.3. Cathelicidins

Cathelicidins are amphipathic, α-helical linear peptides of typically 12 to 88 amino acids in length [76]. Structurally, cathelicidins comprised of a N-terminal signal sequence, a highly conserved “cathelin” domain and variable residues at C-terminus [77]. The distinct feature of cathelicidins is that these proteins are found as inactive precursors (hCAP18) in neutrophils and only after proteolytic cleavage by proteinase 3, an active fragment is released [78]. In humans, a lone member of cathelicidin family was identified, termed LL-37 (37 residues long with two leucine residues at the N-terminus) [79]. Human LL-37 elicits a vast range of activities including antimicrobial, antibiofilm, anticancer and immunomodulatory properties. It is effective at killing bacteria, fungi and enveloped viruses [51,80]. A recent report suggested that the deficiency of vitamin D and air pollution could reduce LL-37 expression and increase the severity of COVID-19 [81].

### 3.4. Mechanisms of Antimicrobial Action of HDPs

HDPs are attractive as an alternative to conventional antimicrobials due to their unique mechanism of action [83]. Studies have shown that at certain sites such as the intestinal lumen and the epithelium of the airway, HDPs do not show antimicrobial activity but act as immunomodulators [84,85]. This may be due to the low concentrations of the HDPs; nevertheless, it provides evidence of multifunctional potential [84]. The ability to kill microbes has long been the crux of the excitement relevant to HDP research. Numerous mechanisms have been proposed for HDPs. More specifically, the antimicrobial activity of HDPs can be separated into two categories: membrane targeting and non-membrane targeting [43,86].

#### 3.4.1. Membrane Targeting Mechanism

The bacterial cytoplasmic membrane mainly composed of anionic lipids: lipopolysaccharides (LPS) in Gram-negative bacteria and teichoic acids in Gram-positive bacteria [87]. These negatively charged constituents in membrane make the bacteria readily available to interact with the positively charged HDPs [88]. In addition, the amphipathic nature of HDPs allows interaction with the phospholipid bilayer of the bacteria, which eventually de-stabilises the bacterial membrane and leads to lytic cell death [89]. Following are the four models proposed for the antimicrobial mechanism: the toroidal pore model, barrel-stave model, carpet-like model and aggregate model [90].

#### 3.4.2. Intracellular Targeting Mechanism

Some HDPs are able to identify and act on intracellular targets [91]. They can disrupt protein biosynthesis by preventing transcription, translation and assembly of proteins [92]. More specifically, HDPs can interfere with chaperone enzymes, which are crucial for correct folding and assembling of newly synthesised proteins [93]. Further studies have shown that HDPs can also interfere in the mechanisms involved in refolding misfolded proteins by permanently closing crucial regions of the protein on which enzymes bind to refold the protein [94]. One such HDP is Tur1A, which inhibits protein biosynthesis in *Escherichia coli* and *Thermus thermophilus* bacteria [95]. As a result of this interference in the protein synthesis machinery, HDPs can prevent the multiplication of the microbe.

HDPs can also inhibit key enzymes involved in the synthesis of nucleic acids within the bacterial cell. In addition, they can trigger DNA topoisomerase I, which results in double-stranded DNA breaks and, therefore, the DNA replication [96]. Studies with a HDP, TFP 1-1TC24 (Tissue factor pathway inhibitor), have shown that HDPs can directly rupture cell membranes and degrade both DNA and RNA [97]. Protease enzymes are also targets of HDPs and their inhibition can interfere with crucial metabolic pathways within bacterium [98]. For example, Histatin 5 shows strong inhibitory effect on proteases secreted by the bacteria, resulting in a dysfunctional bacteria cell [98].

HDPs also play a pivotal role in nutritional immunity against pathogens. They can sequester essential metal ions which weakens the bacterial defence and make them susceptible to the host immune system. One such example is Psoriasin, also referred to as S100A7, which sequesters zinc ions (Zn^2+^), therefore starving the bacteria from crucial metal ions needed in normal functioning [99]. Similarly, another family member of S100A HDPs, Calprotectin, which is also known as S100A8/A9, limits bacteria from manganese ions (Mn^2+^) [100]. As a result, the bacteria’s Mn^2+^-dependent defences were starved and therefore the bacteria become susceptible to neutrophils [100].

As such, even looking at from an antimicrobial point of view, it is evident that by targeting or penetrating the cytoplasmic membrane of the microbe will not always result in the death of the cell. Therefore, interacting and interfering with the microbe’s crucial processes, which are needed for proper functioning, is also an effective approach in preventing infection. Nevertheless, it is crucial to not only destroy or inhibit the microbe but also limit its effects on the body.

### 3.5. Non-Antimicrobial Actions of HDPs

HDPs are multifunctional and their immunomodulatory activity has specifically drawn significant attention [51]. From the earliest studies looking at HDPs on their effects on immune cells, specifically leukocytes, to more recent studies, it has been shown that the understanding of the immunity-related functions of HDPs is increasing exponentially [101,102]. As such, it is known that the mechanisms underpinning the ability of HDPs to modulate immune cells are highly complex, involving intracellular uptake of peptides through G-coupled protein receptors and activation of downstream signalling pathways [103,104]. At low concentration, HDPs can induce the release of chemokines, notably IL-8 and MCP1, which exhibit direct chemotactic activity towards immune cells such as monocytes, neutrophils, dendritic cells, memory T cells, mast cells and macrophages [105,106]. Once these immune cells are at the site of infection, HDPs can enhance the ability of the immune cells, such as the enhancing the ability of phagocytes, to ingest the HDP-opsonised microbial cell, or degranulate immune cells such as mast cells [107,108]. For instance, LL-37 was shown to directly chemoattract neutrophils and eosinophils by interacting with formyl-peptide receptors [109]. HDPs also induce the expression of antiapoptotic proteins, more specifically caspase-3, and therefore interfere in the apoptosis of neutrophils [110,111]. Research has also shown that HDPs may indirectly kill microbes through neutrophil-derived extracellular traps (NETs) or mast cell-derived extracellular traps (MCETs) [112]. Once triggered, the extracellular trap catches and disarms the pathogen [113].

HDPs are also an important link between the innate and adaptive immune systems through induction and maturation of key antigen-presenting cells such as dendritic cells (DCs) [114]. They can promote DC differentiation from haemopoietic precursor cells and monocytes [115]. Eventually, DCs then activate T and B lymphocytes and result in a robust adaptive immune response against the invading pathogen. HDPs can also directly regulate T and B lymphocytes. A previous study demonstrated that the cathelin-related antimicrobial peptide (CRAMP), a murine homolog of human cathelicidin LL-37, increases production of IgG1 by B lymphocytes as a result of suppressed production of IL-4 by T lymphocytes [116].

HDPs have the dual ability to mediate both pro- and anti-inflammatory responses [117]. LL-37 can directly bind anionic LPS on the cytoplasmic membrane of Gram-negative bacteria, resulting in suppression of pro-inflammatory tissue damage [118]. Not only this; LL-37 has shown to suppress key pro-inflammatory molecules such tumour necrosis factor (TNF)-α, IL-1β, TNF-α-induced protein-2 and nitric oxide that were triggered in response to the microbial infection. Notably, LL-37 does not suppress the production of chemokines responsible for immune cell recruitment [119,120,121]. Due to the modulation of both pro- and anti-inflammatory pathways, it can be suggested that HDPs promote immune homeostasis without excessive harmful inflammation [122].

HDPs also play a crucial role in wound healing by inducing chemotaxis of epithelial cells and promoting the production of molecules that are responsible for the restructuring of the extracellular matrix, notably metalloproteinases [123]. Studies have shown that the deficiency of LL-37 can delay wound healing in ulcers, as a result of impaired re-epithelialisation [124]. The exact mechanism for this activity has yet to be deciphered. However, it is proposed that HDPs interact with epidermal growth-factor receptor (EGFR) which undergoes phosphorylation, leading to migration of keratinocytes [125].

## 4. Use of HDPs for Combat against Antimicrobial Resistance

HDPs have been proposed to combat the rising threat of AMR through complete replacement or synergism with conventional antimicrobials. One of the key causes of increased AMR is the over-use of antimicrobial drugs. Due to various geo-political, economic and social factors, it is challenging to implement a change in the global practise, especially the reduction of antimicrobial use. However, studies carried out in veterinary medicine has shown a positive trend towards the reduction of AMR. A report carried out by the European Surveillance of Veterinary Antimicrobial Consumption (ESVAC), which looked at the figures for antibiotics sales and use in veterinary practise, showed that there was an overall decrease of 20% in AMR as 25 countries reduced their antimicrobial consumption [126]. A national plan to decrease antimicrobial consumption was initiated by France and led to a 39% decrease over a six-year period. Further studies were carried out and reported that the proportion of resistant *Escherichia coli* drastically fell from 16% to <2% [127]. Although these results are corroborated by many other studies, they are limited only to veterinary medicine and have not been carried out in human populations, therefore more results are required. However, the positive trend of reduced consumption of antibiotics results in reduced AMR is demonstrated. As such, one may posit the following question: by reducing antimicrobial usage what therapy can be used to prevent microbial infections?

HDPs can target hard-to-treat bacteria due to their innate electrostatic attraction with the anionic bacterial membrane [128]. Alongside this, HDPs have a clear advantage over conventional antibiotics in regard to their broad spectrum of activity, role in immunomodulation and anti-inflammatory response and slower emergence of bacterial resistance [129]. HDPs have also demonstrated microbicidal abilities against enveloped viruses, fungi, and parasites. The antiviral activity of HDPs has been demonstrated in many studies; notably, it can be used for the treatment of infection by human immunodeficiency virus (HIV) [130]. HBD-2 and HBD-3 are induced during HIV infection and are shown to block viral replication by neutralising HIV virions [131]. LL-37 along with many other HDPs have shown to inhibit *Candida albicans* [132]. HDPs such as α-Defensin HNP-1 has shown potent activity against promastigotes and amastigotes forms of the parasite *Leishmania major* [133]. As such all these studies verify the broad-spectrum use of HDPs and therefore HDPs can act as alternatives to antimicrobial use, consequently reducing the consumption of antimicrobials and limiting AMR.

Another way in which HDPs can curb the rise in AMR is due to its insusceptibility or slower emergence in microbial resistance. It is postulated that due to HDPs co-evolving alongside microbes for millions of years and unlike current antibiotics, the acquisition of antimicrobial resistance to HDPs is improbable [134]. However, recent studies have shown that microbes can develop resistance against some natural HDPs. The initial step to resist HDPs is to prevent their attachment to bacterial membrane by changing the membrane components and therefore changing the overall membrane charge [135]. The microbe can do this by increasing expression of amino acylated phospholipids on its membrane resulting in a decreased negative membrane charge, and therefore positively charged HDPs are less readily able to attach and interact with the microbial cell [135]. Many studies have demonstrated this mechanism, one being the study carried out by Li et al. that found that *Staphylococcus aureus*, through its sensory system GRaRS, was able to sense HDPs in the environment and induce D-alanylation of teichoic acid on its membrane, resulting in reduction of membrane charge [136]. Another proposed mechanism for resistance is bacterial efflux pumps actively transporting HDPs out of the cell [137]. This role plays an important part in the resistance mechanism of bacteria to antibiotics and HDPs. These crucial mechanisms do make it possible for resistance to occur; however, one must think that over the millions of years of HDPs and microbes have co-evolved alongside each other, why have microbes not been more successful in resisting the activity of HDPs? The answer lies in the fact that for the microbe to resist the electrochemical attraction of the HDPs, it must modify its entire membrane, resulting in a substantially higher fitness cost [134]. This is unlike antibiotic resistance whereby the bacteria alter only one site, notably the targeted binding site [138].

Other contributors to resistance are the release of proteases by the microbial cell [139]. Proteases can induce proteolytic degradation of HDPs, therefore nullifying their overall effect. There have been many known proteases that degrade HDPs with common examples such as SpeB, which is secreted by *Streptococcus* spp, and V8 protease, which is secreted by *Staphylococcus aureus* [135]. However, the mechanism involved in degrading HDPs is highly dependent on the structure of targeted peptide [140]. Most studies carried out on the proteolytic degradation of HDPs have been on cathelicidin LL-37, which has a linear structure, therefore making them susceptible to degradation. However, studies carried out on non-linear HDPs such as lantibiotics and defensins, which have structures containing a disulphide bond, have shown less susceptibility to degradation by proteases [141]. Thus, HDPs can evolve and form mechanisms to resist degradation. Another approach to circumvent the proteolytic degradation and increase antimicrobial potency is to biochemically modify the structure of HDP variants [50].

Therefore, acquisition of resistance by microbes to HDPs is possible; however, it is improbable. Yes, model studies have shown that bacteria do possess the mechanism required to become resistant to HDPs; however, in organisms, the complexities and multifunction of HDPs say otherwise. Nevertheless, with the passing of time and the natural course of evolution taking place, microbes may acquire resistance. Therefore, disregarding any resistance to HDPs is unsuitable as data using HDPs over a prolonged time is required [142].

Another approach to combating AMR is using HDPs in conjunction with antibiotics [143]. HDPs used as adjuvants can be categorised as class 1 and class 2 adjuvants, with the former being able to actively inhibit antibiotic resistance and the latter enhancing the ability of the host’s immune system to neutralise the microbe. HDPs can display both activities due to their synergistic effect with some antibiotics as well as their ability to immunomodulate (see Table 6). A study carried out by our team tested the effect of a synthetic short derivative of LL-37, namely FK16, in combination with vancomycin against *Pseudomonas aeruginosa*. Vancomycin is usually not preferred for the treatment of Gram-negative infections due to their lack of ability to cross the outer membrane of bacteria. Our results showed that the FK16 makes the OM porous, which allowed vancomycin to penetrate and affect the bacterial fitness causing cell death [144]. Continuing on, our group has recently developed a library of novel bactericidal peptides through hybridisation of two different HDPs sequences, i.e., combining the benefits of two classes of HDPs into one molecule. A hybrid derivative of LL-37 and HBD-3, termed CaD23, was shown to enhance the potency of amikacin against *Staphylococcus aureus* [145,146]. Overall, antimicrobials targeting resistant pathogens will have their effect enhanced by HDP adjuvants and so reducing the effect of AMR.

## 5. Use of HDPs as Potent Antibiofilm Agents

Biofilms are the communities of microorganisms encased within a slimy extracellular matrix. Microbes within the biofilm are usually 1000-fold more resistant to antimicrobial agents than those in a planktonic state [147]. In addition, microbes living in biofilms are highly resistant to environmental stress and innate immune cells. This unique adaptation of biofilm cells poses an enormous challenge in clinical settings [148]. With the growing crisis of AMR and considering that up to 65% of bacterial infections are caused by biofilms, there is an unmet medical need for alternative antimicrobial agents that are capable of targeting biofilms. There are numerous in-depth reviews and expert commentaries reporting the potential of HDPs as antibiofilm therapeutics [42,147,148,149]. Here, rather than duplicating the known facts, we would like to highlight only the key aspects of HDPs for the prevention and management of biofilm infections.

Both natural and synthetic HDPs exert the antibiofilm effects at lower concentrations than those required to kill bacteria in planktonic state. This is because the antibiofilm mechanisms of action of HDPs varies from its widely known membrane-perturbing activity [148]. HDPs can act at one or multiple stages of biofilm formation i.e., bacterial cell-attachment, maturation, and dissemination. In addition, some HDPs are also capable of eradicating preformed biofilms at sub-killing concentrations [148]. Therefore, both prevention and eradication of biofilms are essential in clinical settings and with multiple biological activities, such as wound-healing and immune modulation, HDPs are promising therapeutics to combat AMR and biofilm infections. LL-37, HBD-2 and -3 and Indolicidin were shown to inhibit *P. aeruginosa* biofilms at sub-killing concentrations [147]. LL-37 explicitly promotes the twitching motility of bacteria, which directly impacts the biofilm maturation and increases disassembly [150]. Microarray analysis showed that LL-37 treatment inhibited quorum-sensing signalling mechanisms of P. aeruginosa, which are important for both biofilm formation and maturation. Synthetic HDPs such as cec-4 and 1037 demonstrated a similar antibiofilm activity against Gram-negative pathogens [151,152]. On the other hand, a HDP derived from fish, piscidin-3, was shown to degrade the biofilm matrix by binding cationic copper, causing cleavage of bacterial DNA [153]. Nisin A, a bacteriocin, was shown to disrupt MRSA membrane potential and ATP efflux within the biofilm. Notably, the peptide concentration required to disrupt MRSA in biofilm was 2-10 times higher than those required to kill them in planktonic state [154]. This suggests that the antibiofilm effects of HDPs are diverse, species-specific and peptide-sequence dependent.

Fungal co-infections in severe COVID-19 patients have been widely associated with the increased use of dexamethasone, which was mainly used for the management of cytokine storm [155,156]. Fungi/yeast biofilms are the frequent causes of pulmonary aspergillosis and catheter-induced candidemia. Invasive pulmonary aspergillosis (IPA) is usually difficult to diagnose and manage with available antifungal agents. With the emergence of azole-resistant *Aspergillus spp.*, the morbidity and mortality rates of IPA have exponentially increased [157]. Natural HDPs such as derivatives of LL-37 and histatins have shown potent antibiofilm properties against yeast biofilms in in vivo and in vitro model systems [149]. HDPs derived from venoms of bees (e.g., lasioglossin-III) [158]., wasps (e.g., protonectin) [159] and spiders (e.g., lichosin-1) [160] exert potent antibiofilm activities against *Candida spp.* biofilms. Synthetic HDPs such as IDR-1018 were also shown to be effective in an *in vivo* model of candidemia [161].

HDPs synergy with antibiotics against biofilms have been demonstrated using in-vivo abscess model systems [162]. Although the mechanism of synergy between antibiotics and HDPs against biofilms is not completely understood, it is conceivable that the dispersion of biofilms by HDPs may expose bacteria to antibiotics and immune cells (e.g., neutrophils) and, alternatively, HDPs directly perturbing bacterial membrane may increase antibiotic penetration into bacteria causing cell death. Any future improvements of HDP-antibiotic synergism against biofilm infections are dependent on peptide sequence, which could be optimised utilising structural-activity relationship approaches. Overall, HDPs have great potential to be used as antibiofilm peptide therapeutics and may be championed for the prevention of AMR and eradication of infectious diseases in humans, animals and plants where traditional antibiotic therapies have failed.

## 6. Application of HDPs for the Management of COVID-19

Structural studies led to the discovery that SARS-CoV-2 utilises the receptor-binding domain (RBD) of spike protein for interaction with the angiotensin-converting enzyme 2 (ACE2) on host cell membrane for invasion [163,164]. This discovery spurred the development and testing of multiple therapeutic approaches to inhibit the CoV-2 and ACE2 interactions. Natural HDPs can halt the progression of viral infections utilising one of the known mechanisms, i.e., disruption of the viral envelope, inhibition of DNA replication of virions and blocking the entry of virions into the host cells [165]. An alpha defensin, HD-5, released from the crypts of the small intestine can bind ACE-2 on enterocytes. Wang and co-workers reported that HD-5 can inhibit spike-protein-expressing pseudovirions from binding ACE-2 on enterocytes, ultimately preventing CoV-2 infection [166]. In silico docking studies predicted that LL-37, theta-defensins and HBD-2 can bind the RBD of SARS-CoV-2 [167,168,169]. Biochemical analyses further validated that HBD-2 indeed has strong affinity towards RBD, which prevented the fusion of S-protein expressing pseudovirions with ACE-2 on human cells [169]. Furthermore, intranasal administration of LL-37 was shown to protect mice from respiratory infection caused by spike-protein-expressing pseudovirions [168]. Brilacidin is a novel mimetic of defensin, which is also known as PMX30063, which has been fast tracked by the Federal Drug Administration (FDA) for the treatment of oral mucositis [170]. A recent study has demonstrated the potent antiviral activity of Brilacidin against SARS-CoV-2. It prevents viral entry into the hosts cells by interacting with ACE2 receptors and disrupting viral integrity [171]. HDPs can also be used in combination with other antiviral treatments. Drugs such as remdesivir have shown effectiveness in reducing the recovery time of COVID patients and reduce the risk of further progression [172]. Bakovic et al. demonstrated the efficacy of combining remdesivir with Brilacidin and the data showed that the inhibition profile of this combination treatment was greater than using both compounds alone; notably, a synergistic inhibition of >99% was reported [171]. Currently, Brilacidin is being tested under phase II clinical trials for the treatment of SARS-CoV-2 infection (clinicaltrials.gov identifier: NCT04784897).

The severity and mortality rates of COVID-19 are associated with the increased levels of inflammatory mediators, which are widely referred to as cytokine release syndrome (CRS) [173]. HDPs can dampen key pro-inflammatory molecules such as tumour necrosis factor (TNF)-α, IL-1β, TNF-α-induced protein-2 and nitric oxide. Therefore, they could be an effective utility for the reduction of CRS and lethality of COVID-19 infections. Recent studies have reported that the SARS-CoV-2 virus can overwhelm the innate immune system by supressing defensins and LL-37 [174,175], hence showing the importance of HDPs in guard against microbial infections.

HDPs can be used as adjuvants for the development and use of vaccines [176]. One of the roles of HDPs is to induce the recruitment of antigen-presenting cells (APCs) such as dendritic cells which recognise the antigen on the microbe and initiate an immune response [177]. By using HDPs alongside the vaccine vector, APCs can be readily recruited to the site of vaccine and therefore provide rapid protection against the microbe [178]. Similarly, this method can be used for improving the effectiveness of COVID-19 vaccines.

The above-mentioned preclinical and clinical studies provide proof-of-principle to utilise HDPs for reducing dependency on conventional antimicrobial agents in the management of COVID-19 and associated secondary infections. Further studies are warranted to test the efficacy and safety of HDPs for the treatment of co-infections associated with COVID-19.

## 7. Other Applications of HDPs

HDPs have shown a use in preventing post-surgical infection, including burns and chronic wound infection. PXL150 and D2A21 shown to treat burn wounds in mice, and these are now being tested in clinical trials for treating burn wounds in humans [179]. Additionally, HDPs were also shown to prevent bacterial biofilm development on coated invasive catheters and can be used as topical application to aid in ulcer healing, specifically in diabetic patients [180,181].

In ophthalmology, the use of HDPs as a novel alternative to antimicrobials is welcomed due to increasing numbers of resistant bacteria. Even though HDP use in ophthalmology is in theoretical stages, Lactoferrin B and Protegrin-1 have shown promise in preventing corneal infections [182]. Our group has fully characterised a range of HDPs on human corneal surface during disease and health [50,68,69,70,71,72,183,184,185,186]. Recent studies from our laboratory showed that synthetic HDPs can be utilised for the treatment of Gram-positive and Gram-negative corneal infections [144,145,146].

Research into enhancing the antimicrobial ability of HDPs without increasing cytotoxicity has led to optimisation in the form of synthetic peptides, innate defence regulator peptides (IDR peptides). IDRs are synthetic cationic peptides derived from natural HDPs but have been optimised, enhancing the HDPs immunomodulatory activities [187,188]. IDRs show modest antimicrobial activity but have profound anti-inflammatory activity, as established by various studies. One such study looked at three IDRs: IDR-HH2, IDR-1002 and IDR-1018 [189]. Although IDRs showed a modest antimicrobial activity against *Mycobacterium tuberculosis*; there was considerable reduction in lung inflammation as revealed by decreased pneumonia.

## 8. Limitations of HDPs

As discussed, HDPs have great potential in answering the call to limit AMR and being used as an alternative to antibiotics; however, due to inherent limitations, only 76 HDPs are in the clinical phase of drug development [52].

### 8.1. Cost of Synthesis

One of the key limitations of HDP use is their cost of production. Using common peptide and chemical synthesis techniques such as fluorenylmethyloxycarbonyl (FMOC) chemistry can be expensive to produce synthetic HDPs, which could range from USD 50–400 per gram [190]. One way of tackling this cost would be to produce short and truncated synthetic peptides which mimic the effects of natural HDPs [191]. Computer-aided quantitative structure–activity relationships (QSAR) analysis can be utilised to produce models to test the interaction of the synthetic HDP as well as biological activity [187,192,193]. The QSAR optimisation can streamline the development of cost-effective, short peptides for clinical use [191,194].

### 8.2. Toxicity

Another limitation of HDP use is the development of unknown toxicities. Due to the multifunctional activity of HDPs, certain activities are unpreferred such as histamine secretion from mast cells promoting inflammation and unwanted toxicity against host cells. One such example is gramicidin S, a HDP isolated from the bacteria *Bacillus brevis*, which is restricted to use as a topical antibiotic due to its unwanted haemolytic activity [195]. Further studies have shown that excess levels of LL-37 have an association with psoriasis and rosacea [196,197,198]. As such, HDPs should first be tested against ex vivo human cell cultures at early stages so that HDP toxicity can be minimised and formulations can be optimised. Moreover, QSAR approaches can be utilised to develop synthetic HDP derivatives that are less toxic towards host cells.

### 8.3. Instability

As mentioned previously, HDPs can be susceptible to proteolytic degradation either by the hosts’ internal environmental factors or by the activity of proteases. To enhance stability, HDPs can be modified and synthesised by making more complex structures with the inclusion of disulphide bridges to prevent proteolytic degradation; however, this will inevitably increase the cost of production, which is already a concern and an obstacle in large-scale HDP production [199]. Nano-formulations can prevent the proteolytic degradation and reduce the physiological instability of synthetic HDPs.

With all the above limitations, developing HDPs as clinical therapeutics is a challenge. HDPs have failed at different levels of the clinical stages and no single HDP has been approved for use for systemic infections. One reason for failing at the development stage is that the efficacy of HDPs in vitro and in animal studies show promise; however, it does not translate to efficacy in human populations [200]. Another reason is that HDPs must show improved activity over drugs, specifically antimicrobials. Pexiganan is a synthetic peptide derivative which, irrespective of its relative low toxicity and preclinical efficacy, has been rejected by the Food and Drug Administration (FDA) due to being less effective than available therapies [201]. Therefore, as drugs progress through the development phase, the focus shifts from safe drug dosages to drug efficacy, leading to many failed applications and non-progression, as was the case for Omiganan and Surotomycin [202,203]. Other HDPs failed in this late stage due to increased mortality and renal toxicity for Talactoferrin and Murepavadin, respectively [204,205].

## 9. Conclusions

The field of nanotechnology and artificial intelligence (AI) is growing rapidly, and consideration is being given to how these field can benefit the usage of HDPs. By using nanotechnology as a way of delivering HDPs into the host, resistance mechanisms expressed by the bacteria, such as proteases, can be counteracted. One such delivery system is the nano-polymer PLGA, which has successfully demonstrated the delivery and sustained release of cathelicidin LL-37 [206]. Another approach is to form biopolymers by conjugating HDPs into the nano-polymer and using this biopolymer to create catheters and other invasive medical instruments, resulting in reduced AMR [207]. The introduction of AI in the field of medicine has led to an ease in identifying potent peptide candidates and novel peptides, paving the way for future development [208].

With the rapid decline of the effectiveness of our antimicrobial armamentarium, owing to years of misuse and overuse, we are amidst two pandemics. Before the outbreak of SARS-CoV-2, dealing with the exponentially rising cases of AMR demanded swift global action but now this is complicated, and our priorities are askew. Without alternatives to antimicrobials, the pandemic of AMR will snowball out of control. This review paper has highlighted the answer to both pandemics, i.e., providing potential therapeutic utility of HDPs. These antimicrobial and immunomodulatory peptides are a Jack of all trades, but nay; with optimisation and modification into synthetic molecules, HDPs can also be a master of both. With their effective targeting of microbial membranes and multifunctional activity in modulating the body’s innate immune system, HDPs show promise as an alternative to antimicrobials. As with all microbial-targeting therapies, there is a risk of acquisition of resistant mechanism to HDPs as acquired resistance is a natural evolutionary process, therefore nothing is truly completely resistant. However, due to the high fitness cost of resistant mechanisms against membrane acting HDPs in pathogens, resistance is improbable to perceive. HDPs can also fulfil roles in many non-infective pathologies. All this success can be overshadowed by the harsh reality of zero-approved HDPs in drug development for systemic infections. Many of the HDPs in clinical trials have been withdrawn due to cytotoxicity or lack of efficacy in vivo. However, with better understanding of HDPs, techniques resulting in synthetic peptides, novel approaches and future application are showing promise. Therefore, to truly answer AMR in the era of the COVID-19 pandemic, further research and effort must be carried out to get HDPs out of drug development and into therapeutics.

Method of Literature Search: Electronic databases, including MEDLINE (January 1950–December 2021) and EMBASE (January 1980 to December 2021), were searched for relevant articles related to keywords such as “host defence peptide”, “antimicrobial peptide”, “COVID-19”, “alternative therapy”, and “antimicrobial resistance” were used. Only articles published in English were included. Bibliographies of included articles were manually screened to identify further relevant studies. The final search was updated on 20 December 2021.

## Figures and Tables

**Table 1:** SARS-CoV-2 severity and clinical manifestation.

Severity	Clinical Symptoms
Mild	Fever Cough No radiological abnormalities
Moderate	Pneumonia
Severe	Respiratory distress—respiratory rate ≥30/min Oxygen saturation ≤93%
Critical	Acute Respiratory Distress Syndrome (ARDS)—use of mechanical ventilation Multi-organ failure

**Table 2:** NICE Guidelines for Antibiotic use for people older patients aged 18 and older with suspected community-acquired pneumonia (adapted from ref. [19]).

Empirical Treatment	Antibiotics	Dosage ^1^
Oral antibiotics for moderate or severe pneumonia	Options include:	
	Doxycycline	200 mg on first day, then 100 mg once a day
	Co-amoxiclav and Clarithromycin	500 mg/125 mg three times a day 500 mg twice a day
	In severe pneumonia and if the above options are unsuitable:	
	Levofloxacin	500 mg once or twice a day (consideration given to the safety issues with fluoroquinolones)
Intravenous antibiotics for moderate or severe pneumonia	Options include:	
	Co-amoxiclav and Clarithromycin	1.2 g three times a day 500 mg twice a day
	Cefuroxime and Clarithromycin	750 mg three times a day ^2^ 500 mg twice a day
	In severe pneumonia and if the above options are unsuitable:	
	Levofloxacin	500 mg once or twice a day (consideration given to the safety issues with fluoroquinolones)

^1^ Oral dosage is for immediate release medicines; ^2^ Increased to 750 mg four times a day or 1.5 g three or four times a day if infection is severe.

**Table 3:** NICE Guidelines for Antibiotic use for people older patients aged 18 and older with suspected hospital-acquired pneumonia (adapted from ref. [19]).

Empirical Treatment	Antibiotics	Dosage ^1^
Oral antibiotics for non-severe pneumonia when there is not a higher risk of resistance	Options include:	
	Doxycycline	200 mg on first day, then 100 mg once a day
	Co-amoxiclav	500 mg/125 mg three times a day
	Co-trimoxazole	960 mg twice a day ^2^
	If other options are unsuitable:	
	Levofloxacin	500 mg once or twice a day (consideration given to the safety issues with fluoroquinolones)
Intravenous antibiotics for severe pneumonia (for example, symptoms or signs of sepsis or ventilator-associated pneumonia) or when there is a higher risk of resistance	Options include:	
	Piperacillin with tazobactam	4.5 g three times a day, increased to 4.5 g four times a day of infection is severe
	Ceftazidime	2 g three times a day
	If other options are unsuitable:	
	Levofloxacin	500 mg once or twice a day (consideration given to the safety issues with fluoroquinolones)
Antibiotic to be added if methicillin-resistant Staphylococcus aureus infection is suspected or confirmed (dual therapy with an intravenous antibiotic listed above)	Vancomycin	15 mg/kg to 20 mg/kg two or three times a day intravenously, adjusted according to serum vancomycin concentration. Maximum 2 g per dose ^3^
	Teicoplanin	Initially 6 mg/kg every 12 h for 3 doses intravenously, then 6 mg/kg once a day ^3^
	Linezolid	600 mg twice a day orally or intravenously (with specialist advice only) ^2^

^1^ Oral dosage is for immediate release medicines; ^2^ see the BNF for information on monitoring of patient parameters; ^3^ see the BNF for information on patient parameter and therapeutic drug monitoring.

**Table 4:** Increased dose of antibiotic classes and their dosage-related adverse effects (adapted from ref. [34]).

Antibiotic Class	Dose-Related Adverse Effects
β-lactams	Hepatotoxicity Neutropenia Encephalopathy Transient increase in liver enzymes Neuronal excitation Myoclonus Seizures
Cephalosporins	Neutropenia Nephrotoxicity Neurotoxicity Tonic–clonic seizures
Carbapenems	Neurotoxicity Seizures
Fluoroquinolones	Cardiovascular disorders Tendinopathy Phototoxicity Neuropathy Hepatotoxicity
Macrolides	Cardiac toxicity Gastrointestinal disturbances Hepatotoxicity Ototoxicity
Glycopeptides	Nephrotoxicity Phototoxicity Auditory nerve damage Stevens-Johnson Syndrome (SJS)
Aminoglycosides	Ototoxicity Nephrotoxicity Interference with mitochondrial respiration, protein synthesis and sodium–potassium pump
Polymyxins	Neurotoxicity Nephrotoxicity

**Table 5:** Amino acid sequence and source of common human HDPs (adapted from ref. [82]).

Name	Amino Acid Sequence	Source
HNP-1	ACYCRIPACIAGERRYGTCIYQGRLWAFCC	Neutrophils Bone marrow
HNP-2	CYCRIPACIAGERRYGTCIYQGRLWAFCC	Neutrophils Bone marrow
HNP-3	DCYCRIPACIAGERRYGTCIYQGRLWAFCC	Neutrophils Bone marrow
HNP-4	VCSCRLVFCRRTELRVGNCLIGGVSFTYCCTRV	Neutrophils
HD-5	ATCYCRTGRCATRESLSGVCEISGRLYRLCCR	Paneth Cells (intestinal epithelium) Female reproductive system
HD-6	AFTCHCRRSCYSTEYSYGTCTVMGINHRFCCL	Paneth Cells (intestinal epithelium)
hBD-1	DHYNCVSSGGQCLYSACPIFTKIQGTCYRGKAKCCK	Kidney Skin Salivary glands
Histatin 1	DSHEKRHHGYRRKFHEKHHSHREFPFYGDYGSNYLYDN	Saliva
Histatin 3	DSHAKRHHGYKRKFHEKHHSHRGYRSNYLYDN	Saliva
LL-37	LLGDFFRKSKEKIGKEFKRIVQRIKDFLRNLVPRTES	Neutrophils Skin

**Table 6:** Synergism between HDPs and antibiotics (adapted from ref. [143]).

HDPs	Synergistic Interaction with Antibiotics	Microorganism
Cryptidin-2	Ampicillin	*Salmonella typhimurium*
Arenicin-1	Ampicillin	*Staphylococcus aureus*
	Erythromycin	*Staphylococcus epidermidis*
	Chloramphenicol	*Pseudomonas aeruginosa* *Escherichia coli*
Nisin Z	Penicillin	*Pseudomonas fluorescens LRC-R73*
	Streptomycin	Penicillin-resistant variant
	Leucomycin	
	Rifampicin	Streptomycin-resistant variant Lincomycin-resistant variant Rifampicin-resistant variant
Nisin	Daptomycin	*Methicillin resistant Staphylococcus aureus* (MRSA) biofilms
Indolicidin	Teicoplanin	
CAMA	Ciprofloxacin	
Nisin	Ampicillin	*Staphylococcus aureus*
	Daptomycin	*Enterococcus faecalis*
	Ampicillin	*Salmonella typhimurium*
	Cefotaxime	
	Ceftriaxone	
Brevinin-2 CE	Levofloxacin	ESBL producing *Escherichia coli*
	Amoxicillin	
	Chloramphenicol	*Methicillin-resistant Staphylococcus aureus* (MRSA)
Human beta defensin 3	Tigecycline	*Clostridium difficile*
Cathelicidin (LL-37)	Moxifloxacin	
	Piperacillin-Tazobactam	
	Meropenem	
	Azithromycin	*MDR Pseudomonas aeruginosa* *MDR Klebsiella pneumonia* *MDR Acinetobacter baumannii*
LL-37 derivatives	Vancomycin, Chloramphenicol	*Pseudomonas aeruginosa*

## Data Availability

Not applicable.
